# Ovariotomy

**Published:** 1868-01-15

**Authors:** 


### *EDITORIAL.*

---

Editorial.—*Several articles crowded out of this number by pressure of other matter.*

Back Nos. Wanted.—*Owing to the large increase in our subscription list, although it was supposed a sufficient number of copies had been provided, we are entirely out of copies of the* Journal *for the months of* January, June, September, October, *and* November, 1867, *Vol.* XXIV. *We will pay twenty-five cents each fcr copies of either of these, in cash or by credit on the present volume, as desired. Will our friends who do not file their numbers oblige us by responding?*

Ovariotomy.—It is somewhat noteworthy that in the cases of abdominal section we read or hear of, the successful ones are those which skilled and intelligent practitioners have given a grave prognosis upon — the unsuccessful have been those in which all have agreed that, if ever, all circumstances favored the operation. The presumed (or assumed) skill of the operator seems to have little to do with the result. The secret of this discrepancy would appear to be coming out in this wise: *The greater the departure of the peritoneum from its normal condition, the less the danger of interfering with it.* It will hence occur that the *adhesions* so much written and talked about, involving loss of mobility of the tumor; long continuance of pressure; frequent resort to paracentesis, etc., so far from discouraging the operation are rather promotive of confidence in its success. Should this idea become general, it would relieve the professional mind of extreme anxiety, and at the same time give confidence in the employment of measures for temporary relief. Paracentesis is not as dangerous as the long section — meanwhile the patient may be kept in a condition of comparative comfort for years. (We know, personally, of one case, which was tapped at gradually decreasing intervals for over *seventeen* years.) And then the operation may be attempted, as a *dernier resort*, with even better hopes of successful issue.

